# Binary Response Analysis Using Logistic Regression in Dentistry

**DOI:** 10.1155/2022/5358602

**Published:** 2022-03-08

**Authors:** Natchalee Srimaneekarn, Anthony Hayter, Wei Liu, Chanita Tantipoj

**Affiliations:** ^1^Department of Anatomy, Faculty of Dentistry, Mahidol University, Bangkok 10400, Thailand; ^2^Department of Business Information and Analytics, University of Denver, Denver, CO 80208-8921, USA; ^3^S3RI and School of Mathematics, University of Southampton, Highfield, Southampton SO17 1BJ, UK; ^4^Department of Advanced General Dentistry, Faculty of Dentistry, Mahidol University, Bangkok 10400, Thailand

## Abstract

Multivariate analysis with binary response is extensively utilized in dental research due to variations in dichotomous outcomes. One of the analyses for binary response variable is binary logistic regression, which explores the associated factors and predicts the response probability of the binary variable. This article aims to explain the statistical concepts of binary logistic regression analysis applicable to the field of dental research, including model fitting, goodness of fit test, and model validation. Moreover, interpretation of the model and logistic regression are also discussed with relevant examples. Practical guidance is also provided for dentists and dental researchers to enhance their basic understanding of binary logistic regression analysis.

## 1. Introduction

Multivariate analysis is extensively used in multidisciplinary research fields, given its ability to explore multiple independent variables [1]. Research on dental science usually investigates the effect of multiple factors associated with various events, such as factors related to a disease or to the success or failure of an intervention. In dentistry, binary response variable is often recorded as a dependent variable, e.g., success-failure of a treatment, presence-absence of a disease, sound-decayed tooth, positive-negative staining, or other yes-no outcomes. Numerous studies also aim to inspect the relationship between a binary response variable and several independent variables.

Binary logistic regression is an existing causes and effects analysis for such binary response variable as the presence or absence of disease in epidemiology study, positive or negative in laboratory research, or even in the sex prediction in forensic identification of anonymous bodies. It is commonly used to investigate an existing problem by exploring associated factors and predicting the response probability for a new case [2]. Many aspects of the logistic regression differ from those of linear regression, although most researchers are familiar with the latter. Hence, understanding the application and interpretation of binary response analysis is of utmost importance to researchers. The aim of this paper is to explain important concepts of logistic regression with relevant examples, including model fitting, goodness of fit test, validation of the fitted model, and interpretation of the fitted model.

## 2. Linear Regression and Logistic Regression

Regression analysis, a common statistical method employed in dental research, is used to investigate the relationship between one response variable and one or more independent variables. Linear regression and logistic regression differ on the response variable: linear regression for continuous response variable and logistic regression for binary response variable as shown in Table 1. Linear regression models a continuous response variable (*Y*) by a linear combination of independent variables (*Xs*), as in equation (1) [3]:

(1) $$\begin{array}{c} Y=\beta_{0}+\beta_{1}X_{1}+\beta_{2}X_{2}+\beta_{3}X_{3}+\cdots , \end{array}$$

where *β*_*i*_ is a regression coefficient for each *X*_*i*_ that can be continuous, discrete, or categorical variables, e.g., to determine the factors (independent variables, *Xs*) associated with salivary glucose levels (a continuous response variable, *Y*) [4]. The response variable for logistic regression is a binary response variable (*Y*=1 or 0), e.g., success, presence, disease, or positive [5].

Logistic regression model is a construction of the relationship between *p*, the probability of an event of interest, *P*(*Y*=1), and a linear combination of independent variables (*Xs*) with the logit link function. The most commonly used link functions are logit, probit, and complementary log-log [6]. The logit link function is the natural log of the odds ratio—the ratio between the probability of occurrence of an event of interest (if occurred  *p*, and if not occurred  1 − *p*) as shown in equation (2) [7]:

(2) $$\begin{array}{c} \text{logit}\left(p\right)=\ln\left(\frac{p}{1-p}\right)=\beta_{0}+\beta_{1}X_{1}+\beta_{2}X_{2}+\beta_{3}X_{3}+\cdots . \end{array}$$

**Figure 1:**
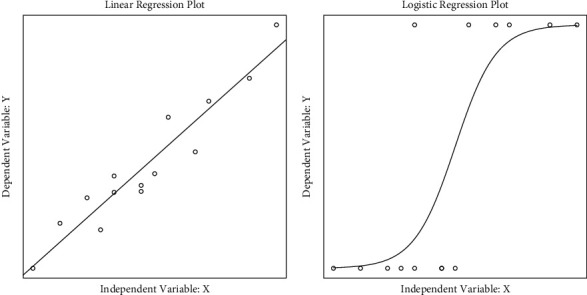
(a) Regression plots from linear regression analysis (left) and (b) logistic regression analysis (right) with one continuous independent variable.

The probit link function is the inverse normal cumulative distribution function. The complementary log-log is the natural log function in terms of log(−log(1 − *p*)) [8]. However, the logit link function is most commonly utilized because it is less complicated and easy to interpret [6]. Therefore, in this paper, only the logit link function of logistic regression will be focused.  Figure 1 shows the regression plots from the continuous response linear regression analysis and binary response logistic regression analysis with one continuous independent variable. The plot from the linear regression analysis is a straight line, whereas for logistic regression, it is a S-curve. The differences are due to the logit link function in logistic regression.

## 3. Sample Size for Logistic Regression

Peduzzi et al. (1996), in a simulation study for sample size in logistic regression analysis, suggested that the number of interested events of a response variable should be at least 10 cases or events per one independent variable [9]. For example, a study on a presenting oral microbe and 5 related factors need 50 cases of the presenting oral microbe. However, if the sample size is limited, Vittinghoff et al. (2007) stated that 5–9 events per variable with bootstrap resampling validation was acceptable [10]. Likewise, a study of an oral lesion and 5 related factors needs 25–45 cases of the oral lesion for satisfactory analysis with bootstrap validation.

## 4. Fitting Logistic Regression Model

**Figure 2:**
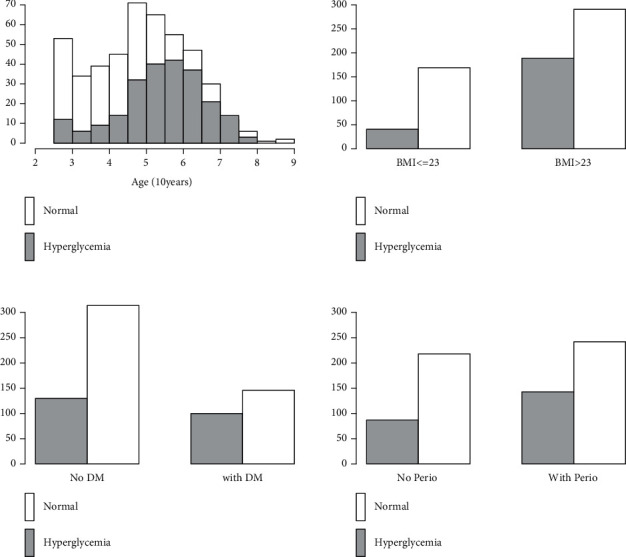
Demographic data of significant variables; age, BMI, family history of DM, and periodontal status, from a study of risk factors associated with hyperglycemia using binary logistic regression analysis [14].

Unlike discriminant analysis, logistic regression does not require the assumption of multivariate normal distribution or any distributional assumption on the *Xs* [11]. The significance of independent variables in the model is determined by the Wald test, which is a proportion between the estimating parameter *β*_*i*_ to its standard error that is assumed to follow a standard normal distribution [12]. Several statistical software programs: SPSS, R, STATA, SAS, etc. present the square of the proportion, which follows a chi-square distribution with 1 degree of freedom [13]. The null hypothesis for both statistical tests is *β*_*i*_=0. The independent variables with a *p* value greater than the significance level is removed from the model. An example of data from a study of risk factors associated with hyperglycemia using binary logistic regression analysis, after removal of a few nonsignificant variables, is presented in Table 2 and Figure 2 [14]. The final fitted model can be written as

(3) $$\begin{array}{c} \ln\left(\frac{p}{1-p}\right)=-3.729+0.371*\text{Age}+0.961*\text{BMI}+0.558*\text{HDM}+0.250*\text{PD}, \end{array}$$

where *p* is the probability that the patient would have hyperglycemia.

## 5. Interpretation of Coefficients

Interpretation of the logistic regression model is based on the exponential function. The exponential function of the logistic regression coefficient is the odds ratio. If the independent variable, *X*_*i*_, is increased by 1 unit, the odds of response would be increased by *e*^*β*_*i*_^, when the other variables are fixed [15]. The exponential function was added to equation (3) and changed into the odds ratio, *p*/(1 − *p*), as

(4) $$\begin{array}{c} \frac{p}{1-p}=e^{-3.729}*e^{0.371*\text{Age}}*e^{0.961*\text{BMI}}*e^{0.558*\text{HDM}}*e^{0.250*\text{PD}}. \end{array}$$

The clinical implication of this mathematical equation can be illustrated as the adjusted odds ratio (OR) in Table 1. If the age of the patient is increased by 10 years (1 unit), the odds of hyperglycemia would be increased by *e*^0.371^ or 1.449, when the other *X* variables were fixed. The coefficient of the categorical variable can be interpreted in a similar way. If the patient has a family history of diabetes mellitus (HDM), the odds of developing hyperglycemia would be increased *e*^0.558^ or 1.747 times that of those without HDM, when the other variables were fixed. Those odds are adjusted by the other variables in the equation and are reported as the adjusted odds ratios [16–18]. The confidence intervals of the regression coefficients are also reported. In some statistical packages: SPSS, R, STATA, SAS, etc., exponential function was taken to those intervals. Then, the confidence interval of the odds ratio was reported instead of confidence interval of the coefficients, as in Table 2 [19].

However, some studies aim to estimate the probability of an event of interest. The odds in equation (4) can be simplified to be the probability equation, as in equation (5):

(5) $$\begin{array}{c} p=\frac{\exp\left(-3.729+0.371*\text{Age}+0.961*\text{BMI}+0.558*\text{HDM}+0.250*\text{PD}\right)\;}{1+\exp\left(-3.729+0.371*\text{Age}+0.961*\text{BMI}+0.558*\text{HDM}+0.250*\text{PD}\right)}, \end{array}$$

where exp(·) is an exponential function, and *p* is an estimated probability of having hyperglycemia, as an interested event occurrence, which was the purpose of that study.

The second example in Table 3 aimed to use logistic regression only for prediction. The study of sex determination using tooth widths established a logistic regression model for predicting the probability of an anonymous dead body for being male, using lower-left canine (LLC) and upper intercanine width (UIW) as independent variables [20]. Table 3 presents the result from logistic regression analysis which can be written as

(6) $$\begin{array}{c} ln\left(\frac{p\left(male\right)}{1-p\left(male\right)}\right)=-20.089+1.592*LLC+0.247*UIW. \end{array}$$

Equation (6) is for sex determination of an unknown dead body. Addition of LLC and UIW into the equation results in a probability of being male, *p*(male), which can be used for sex identification.

Another example in Table 4 aimed to use logistic regression only for investigating the risk factors. The study of the prevalence and risk factors of high-level oral microbe used logistic regression to investigate the risk factors [21]. Table 4 presents the result from the analysis which comprised of the statistically significant risk factors associated with the high-level oral microbe. This example presents the odds ratio of three-level categorical variables, i.e., education level. If there are more than two levels of variables, the table should present all levels to show the reference level as non/primary education level in Table 4. Then, the odds ratio of secondary and higher education level, 5.26 and 1.97, respectively, can be compared to the reference level or non/primary education level in this study. For example, if the participant's education is secondary level, the risk of having high-level oral microbes is 5.26 times compared to the one whose education is non/primary level with 95% confidence interval (range 1.41 to 19.67). However, this study only aimed to investigate the risk factors. Since the adjusted odds ratio was adequate for interpretation, the prediction equation was not necessary for this study.

## 6. Goodness of Fit Test

When the final model is constructed, it should be examined in terms of the goodness of fit to describe how well the model fits the data. The *R*^2^ value of logistic regression is usually low, which is different from the *R*^2^ of linear regression. Hosmer et al. (2013) recommended performing the goodness of fit test instead of reporting the *R*^2^ [12]. The Hosmer–Lemeshow goodness of fit statistic is calculated by the grouping method on percentiles of the estimated probability, which follows chi-square distribution. The null hypothesis of this test will verify the fitting of the model. If the calculated *p* value from the test is less than the level of significance, the model can be assumed to be a poor fit [12, 22]. For example, the fitted model in Table 2 had been tested by the Hosmer–Lemeshow test, with a *p* value = 0.210. This indicated that the model fitted the data well. However, Hosmer et al. (1997 and 2002) found that none of the goodness of fit tests has high accuracy when the sample size is small (*n* = 100). Therefore, they recommended a sample size of 500 for the goodness of fit test [23, 24].

## 7. Model Validation

Validation of the fitted model is to confirm the inference accuracy [25]. Before the model fitting process, all data are split into two sets. The first is a validation set (or testing set) and is taken randomly from about 15 to 40% of all data [26, 27]. The rest of the data is called the training set (or modelling set). It is used for the model establishment with logistic regression analysis, which, in turn, will establish the prediction equation, e.g., as in equation (3). Then, the data from the validation set are applied to the previously fitted model from the training data [3, 26]. Model validation is performed by comparing the results from the fitted model and realistic response [15, 28]. The prediction error can be calculated using the incorrect results from the validation set as percent error. For example, if the results from the validation set of 50 samples contain 10 incorrect predictions, the prediction error will be 20% (10/50).

## 8. Various Applications in Dentistry

Superior to the univariable analysis, multiple logistic regression presents the effect of confounding factors and/or other variables with adjusted odds ratios to confirm the effect of interested variables when other factors are involved. Binary logistic regression can be used not only in the investigation of associated factors, as previously described in the example for evaluating the risk factors associated with hyperglycemia but also in many other aspects, as in Table 5. Logistic regression can be applied to investigate related factors, e.g., studies on factors related to tooth loss, tooth wear, implant failure, or temporomandibular joint clicking [29–32]. It can also be used to establish associations between different variables, e.g., between malocclusion and quality of life, between dentist characteristics and treatment decision, between demographic data and awareness of dental waste management, or between consanguineous marriage and dental carries [33–36]. Logistic regression can be used for developing a predictive model, such as sex identification using oral measurements in forensic science or the prediction of esthetic preference using demographic data [20, 37, 38]. The model can also be applied as a screening test for hyperglycemic patients [39] or identifying the stage of carcinoma using specific genes [40].

## 9. Conclusion

Binary logistic regression is utilized in dental research to understand the relationship between multiple independent variables and a binary response variable. Regression coefficients of a final model can describe the significance of each independent variable in regards to the response variable in terms of the odds ratio and Wald test. Moreover, the established model can predict the probability of a new case with the help of the probability equation. The goodness of the fit test for the final model can be examined by the Hosmer–Lemeshow test. Validation of the model can be carried out by dividing the data into validation and training sets. However, clinical factors must be considered for model plausibility. This review provides practical guidance to dentists and dental researchers alike to enhance their understanding of the analysis, which is greatly beneficial when reading articles or performing clinical research that involves binary response.

## Figures and Tables

**Table 1:** Comparison between linear regression and logistic regression.

Regression	Response variable (*Y*)	Examples
Linear regression	Continuous	Score, saliva flow rate, surface hardness, distance
Logistic regression	Binary	Success-failure, presence-absence, sound-decayed tooth, positive-negative, yes-no

**Table 2:** Results from a study of risk factors associated with hyperglycemia using binary logistic regression analysis [14].

Independent Variables	*B* ^*a*^	SE^*b*^	Wald	*p* value	OR^c^	95% CI of OR^*c*^
Constant	−3.729	0.441	71.646	<0.001	0.024	
Age (10 years)	0.371	0.071	27.484	<0.001	1.449	1.261–1.665
Body Mass index (BMI >23 kg/m^2^)	0.961	0.204	22.176	<0.001	2.614	1.752–3.900
Family history of DM (HDM)	0.558	0.175	10.120	0.001	1.747	1.239–2.463
Periodontal status (PD)	0.250	0.121	4.261	0.039	1.284	1.013–1.629

^*a*^ *B*, regression coefficient (*β*_*i*_); ^*b*^SE, standard error; ^*c*^95% CI of OR, 95% confidence interval of odds ratio (exp  *β*_*i*_).

**Table 3:** Results from a sex determination study by tooth widths using binary logistic regression analysis [20].

Independent variables	*B* ^*a*^	*p* value	Odds ratio	95% CI of OR^*b*^
Constant	−20.089	<0.001		
Lower-left canine (LLC)	1.592	<0.001	4.912	2.315–10.422
Upper intercanine width (UIW)	0.247	0.003	1.280	1.087–1.507

^*a*^ *B*, regression coefficient (*β*_*i*_); ^*b*^95% CI of OR, 95% confidence interval of odds ratio (exp*β*_*i*_).

**Table 4:** Results from a study of prevalence and risk factors of high-level oral microbe using binary logistic regression analysis [21].

Independent variables	*p* value	Odds ratio	95% CI of OR^*a*^
Site: Rural	0.002	14.73	2.65–82.00
Hyposalivation	<0.001	23.00	4.15–127.36
Number of tooth loss	0.041	1.08	1.003–1.17
Education level			
Non/primary	Ref	1	-
Secondary	0.014	5.26	1.41–19.67
Higher	0.339	1.97	0.49–7.86

^*a*^95% CI of OR, 95% confidence interval of odds ratio.

**Table 5:** Application of binary logistic regression in dentistry.

Application in dentistry	Binary response variable	Independent variables	Authors (year)
Related factors	Tooth loss (yes/no)	Related factors	Urzua (2012) [29]
	Temporomandibular joint clicking (yes/no)	Dental malocclusion features	Manfredinia (2014) [30]
	Erosion tooth wear (yes/no)	Daily diet, habit, and health conditions	Kitasako (2017) [31]
	Failure of the implants (success/failure)	Predictive variables	Mayta-Tovalino (2020) [32]

Association	Quality of life (good/poor)	Malocclusion and sociodemographic	Anthony (2018) [33]
	Decision to choose an indirect pulp capping (yes/no)	Demographic data, dentist characteristics	Kakudate (2019) [34]
	Awareness, knowledge, and management of biological waste (correct/incorrect)	Demographic data	Diaz-Soriano(2020) [35]
	Dental carries (yes/no)	Consanguineous marriage and other factors	Khan (2020) [36]

Predictive model	Sex determination (male/female)	Canine and intercanine widths	Keawmuangmoon (2017) [20]
	Sex determination (male/female)	Palatal and incisive papilla morphology	Mustafa (2019) [37]
	Esthetic variation (present/absent)	Demographic data	Rosenstiel (2002) [38]

Screening test (risk score)	Hyperglycemia (yes/no)	Risk factors	Tantipoj (2020) [39]

Identify stage-specific genes	Oral squamous cell carcinoma stage (tumor/normal)	Differentially expressed genes	Randhawa (2015) [40]

## Data Availability

The data used to support the findings of this study are available from the corresponding author upon request.

## References

[1] Katz M. H. (2011). *Multivariable Analysis: A Practical Guide for Clinicians and Public Health Researchers*.

[2] Cox D. R., Snell E. J. (2018). *Analysis of Binary Data*.

[3] Faraway J. J. (2015). *Linear Models with R*.

[4] Sashikumar R., Kannan R. (2010). Salivary glucose levels and oral candidal carriage in type II diabetics. *Oral Surgery, Oral Medicine, Oral Pathology, Oral Radiology & Endodontics*.

[5] Severini T. A. (2011). *Elements of Distribution Theory*.

[6] Faraway J. J. (2016). *Extending the Linear Model with R: Generalized Linear, Mixed Effects and Nonparametric Regression Models*.

[7] Kirkwood B., Sterne J. (2003). *Essential Medical Statistics*.

[8] Myers R. H., Montgomery D. C., Vining G. G. (2010). *Generalized Linear Models: With Applications in Engineering and the Sciences*.

[9] Peduzzi P., Concato J., Kemper E., Holford T. R., Feinstein A. R. (1996). A simulation study of the number of events per variable in logistic regression analysis. *Journal of Clinical Epidemiology*.

[10] Vittinghoff E., McCulloch C. E. (2007). Relaxing the rule of ten events per variable in logistic and cox regression. *American Journal of Epidemiology*.

[11] Harrell F. E. (2015). *Regression Modeling Strategies: With Applications to Linear Models, Logistic Regression, and Survival Analysis*.

[12] Hosmer D. W., Lemeshow S., Sturdivant R. X. (2013). *Applied Logistic Regression*.

[13] Kleinbaum D. G., Klein M. (2010). *Logistic Regression: A Self-Learning Text*.

[14] Tantipoj C., Sakoolnamarka S. S., Supa-amornkul S. (2017). Screening for type 2 diabetes mellitus and prediabetes using point-of-care testing for HbA1c among Thai dental patients. *Southeast Asian Journal of Tropical Medicine and Public Health*.

[15] Weisberg S. (2014). *Applied Linear Regression*.

[16] Humphris G., King K. (2011). The prevalence of dental anxiety across previous distressing experiences. *Journal of Anxiety Disorders*.

[17] Darmawikarta D., Chen Y., Carsley S. (2014). Factors associated with dental care utilization in early childhood. *Pediatrics*.

[18] Yip J. K., Borrell L. N., Cho S., Francisco H., Tarnow D. P. (2013). Association between oral bisphosphonate use and dental implant failure among middle-aged women. *Journal of Clinical Periodontology*.

[19] Allison P. D. (2012). *Logistic Regression Using SAS: Theory and Application*.

[20] Kaewmuangmoon J., Arayapisit T., Srimaneekarn N. (2018). Sex determination using canines in Thais. *The Journal of the Dental Association of Thailand*.

[21] Tantipoj C., Khovidhunkit S. P., Panyayong W. (2020). Prevalence and risk factors of high-level oral microbe among dental patients. *Khon Kaen University Dental Journal*.

[22] Hosmer D. W., Taber S., Lemeshow S. (1991). The importance of assessing the fit of logistic regression models: a case study. *American Journal of Public Health*.

[23] Hosmer D. W., Hosmer T., Le Cessie S., Lemeshow S. (1997). A comparison of goodness-of-fit tests for the logistic regression model. *Statistics in Medicine*.

[24] Hosmer D. W., Hjort N. L. (2002). Goodness-of-fit processes for logistic regression: simulation results. *Statistics in Medicine*.

[25] Kim M., Mallory C. (2020). *Statistics for Evidence-Based Practice in Nursing*.

[26] Rhinehart R. R. (2016). *Nonlinear Regression Modeling for Engineering Applications: Modeling, Model Validation, and Enabling Design of Experiments*.

[27] Haynes R. B., Sackett D. L., Guyatt G. H., Tugwell P. (2006). *Clinical Epidemiology: How to Do Clinical Practice Research*.

[28] Hjorth J. S. U. (1999). *Computer Intensive Statistical Methods: Validation Model Selection and Bootstrap*.

[29] Urzua I., Mendoza C., Arteaga O. (2012). Dental caries prevalence and tooth loss in chilean adult population: first national dental examination survey. *International Journal of Dentistry*.

[30] Manfredini D., Perinetti G., Guarda-Nardini L. (2014). Dental malocclusion is not related to temporomandibular joint clicking: a logistic regression analysis in a patient population. *The Angle Orthodontist*.

[31] Kitasako Y., Sasaki Y., Takagaki T., Sadr A., Tagami J. (2017). Multifactorial logistic regression analysis of factors associated with the incidence of erosive tooth wear among adults at different ages in Tokyo. *Clinical Oral Investigations*.

[32] Mayta-Tovalino F., Mendoza-Martiarena Y., Romero-Tapia P. (2019). An 11-year retrospective research study of the predictive factors of peri-implantitis and implant failure: analytic-multicentric study of 1279 implants in Peru. *International Journal of Dentistry*.

[33] Anthony S. N., Zimba K., Subramanian B. (2018). Impact of malocclusions on the oral health-related quality of life of early adolescents in ndola, Zambia. *International Journal of Dentistry*.

[34] Kakudate N., Yokoyama Y., Sumida F., Matsumoto Y., Gordan V. V., Gilbert G. H. (2019). Dentists’ practice patterns of treatment for deep occlusal caries: findings from a dental practice-based research network. *Journal of Dentistry*.

[35] Diaz-Soriano A., Gallo W., Luza S., Munive-Degregori A., Bocanegra R., Mayta-Tovalino F. (2020). Knowledge and awareness of effective recycling of dental materials and waste management among Peruvian undergraduate students of dentistry: a logistic regression analysis. *Journal of International Society of Preventive and Community Dentistry*.

[36] Khan S. (2020). Inheritance and susceptibility to dental caries: a community-based study. *Journal of International Society of Preventive and Community Dentistry*.

[37] Mustafa A. G., Tashtoush A. A., Alshboul O. A., Allouh M. Z., Altarifi A. A. (2019). Morphometric study of the hard palate and its relevance to dental and forensic sciences. *International Journal of Dentistry*.

[38] Rosenstiel S. F., Rashid R. G. (2002). Public preferences for anterior tooth variations: a web-based study. *Journal of Esthetic and Restorative Dentistry*.

[39] Tantipoj C., Srimaneekarn N., Supa-amornkul S. (2020). Development of a risk score to predict abnormal glycemic status among Thai dental patients. *Journal of Health Research*.

[40] Randhawa V., Acharya V. (2015). Integrated network analysis and logistic regression modeling identify stage-specific genes in Oral Squamous Cell Carcinoma. *BMC Medical Genomics*.

